# Overexpressed Galectin-3 in Pancreatic Cancer Induces Cell Proliferation and Invasion by Binding Ras and Activating Ras Signaling

**DOI:** 10.1371/journal.pone.0042699

**Published:** 2012-08-10

**Authors:** Shumei Song, Baoan Ji, Vijaya Ramachandran, Huamin Wang, Margarete Hafley, Craig Logsdon, Robert S. Bresalier

**Affiliations:** 1 Department of Gastroenterology, Hepatology, and Nutrition, University of Texas MD Anderson Cancer Center, Houston, Texas, United States of America; 2 Department of Cancer Biology, University of Texas MD Anderson Cancer Center, Houston, Texas, United States of America; 3 Department of Pathology, University of Texas MD Anderson Cancer Center, Houston, Texas, United States of America; Wayne State University School of Medicine, United States of America

## Abstract

Pancreatic cancer (PDAC) is a lethal disease with a five-year survival of 3–5%. Mutations in K-Ras are found in nearly all cases, but K-Ras mutations alone are not sufficient for the development of PDAC. Additional factors contribute to activation of Ras signaling and lead to tumor formation. Galectin-3 (Gal-3), a multifunctional β-galactoside-binding protein, is highly expressed in PDAC. We therefore investigated the functional role of Gal-3 in pancreatic cancer progression and its relationship to Ras signaling. Expression of Gal-3 was determined by immunohistochemistry, Q-PCR and immunoblot. Functional studies were performed using pancreatic cell lines genetically engineered to express high or low levels of Gal-3. Ras activity was examined by Raf pull-down assays. Co-immunoprecipitation and immunofluorescence were used to assess protein-protein interactions. In this study, we demonstrate that Gal-3 was highly up-regulated in human tumors and in a mutant K-Ras mouse model of PDAC. Down-regulation of Gal-3 by lentivirus shRNA decreased PDAC cell proliferation and invasion in vitro and reduced tumor volume and size in an orthotopic mouse model. Gal-3 bound Ras and maintained Ras activity; down-regulation of Gal-3 decreased Ras activity as well as Ras down-stream signaling including phosphorylation of ERK and AKT and Ral A activity. Transfection of Gal-3 cDNA into PDAC cells with low-level Gal-3 augmented Ras activity and its down-stream signaling. These results suggest that Gal-3 contributes to pancreatic cancer progression, in part, by binding Ras and activating Ras signaling. Gal-3 may therefore be a potential novel target for this deadly disease.

## Introduction

Pancreatic ductal adenocarcinoma (PDAC) is currently the fourth leading cause of cancer-related death, with an estimated 43,140 new cases and 36,800 deaths in the United States [1]. Because of its aggressive growth, early metastatic dissemination and the lack of effective therapies, the five-year survival rate for this disease remains at 3–5% [2]. Considerable effort has therefore been made to understand the molecular events which may drive the pathogenesis of PDAC. Among the numerous molecular alterations identified in PDAC, mutations in the pro-oncogene K-Ras are found in nearly all cases [3] and is an early event for the development of PDAC [4]. K-Ras mutations alone are not sufficient for the development of PDAC. K-Ras mutations are often found in chronic pancreatitis and may even be found in normal individuals [5]. Moreover, K-Ras mutation in a mouse model with low Ras activity does not spontaneously lead to development of PDAC [6], while K-Ras mutation in a mouse model with a high level of Ras activity is associated with rapid development of CP with abundant fibrosis and progression to PDAC which mimics human disease [3]. It has therefore been proposed that it is the activity of K-Ras rather than the presence of mutation per se which is the biologically relevant parameter associated with the pathogenesis of pancreatic cancer [2]. Additional factors are required that contribute to Ras activity; however, the mechanisms by which Ras activity is further activated are largely unknown.

Galectin-3 (Gal-3), a b-galactoside-binding protein exhibits pleiotropic biological and pathological functions, and has been implicated in cell growth, differentiation, adhesion, RNA processing and malignant transformation [7]–[10]. Gal-3 is found in multiple cellular compartments including the cytoplasm, the cell surface, the nucleus, and Gal-3 is also secreted [11]. The significance of Gal-3 expression has been evaluated in many cancer types including pancreatic cancer [12]–[16]. Several studies have indicated that Gal-3 mRNA is up-regulated in pancreatic tumor tissues compared to control tissues [15], [17], [18], [19], and transient suppression of galectin-3 has been reported to induce pancreatic cancer cell migration and invasion [16]. Wang et al found that Gal-3 was also up-regulated in chronic pancreatitis and suggested that it was involved in both extracellular matrix (ECM) changes and ductal complex formation [20]. However, the full significance of Gal-3 in PDAC remains unclear and little is known about the possible function mechanisms of Gal-3 in the pathogenesis of the PDAC. Recently, Kloog and colleagues demonstrated that K-RAS GTP recruits Gal-3 from the cytosol to the plasma membrane where it becomes an integral nanocluster component. The cytosolic level of Gal-3 determines the magnitude of K-Ras GTP nanoclustering and signal output in breast cancer cells [21]. More recently, observations from same group demonstrated that K-Ras association with Gal-3 contributes to thyroid malignancy [22]. Since mutations in K-Ras are nearly universal in PDAC and the activity level of Ras appears to be a key mechanism controlling the development of PDAC, we sought to determine whether Gal-3 affects Ras activity contributing to the pathogenesis of pancreatic cancer.

In this study, we systematically evaluated the expression of Gal-3 in 120 paired human pancreatic tissues from normal pancreas, pancreatitis and pancreatic tumors, and for the first time determined the expression of Gal-3 in tissues and tumor cells derived from of a mutant K-Ras mouse model of pancreatic cancer. We have extended the results of previous studies, and further delineated the function of Gal-3 in vivo and in vitro with regard to pancreatic cancer formation. Gal-3 expression was increased in pancreatic cancers and cancer cells, and stimulated pancreatic cancer cell proliferation and invasion and promoted tumor growth. Gal-3 binds Ras and enhances Ras activity and down-stream signaling. These observations support the conclusion that Gal-3 may be a potential novel target for this deadly disease.

## Materials and Methods

Animal-related studies have been approved by the M.D Anderson Cancer Center Institutional IACUC committee (ACUF 09-04-08832). All other studies presented herein were the investigator-initiated and did not require approval from other regulatory bodies.

### Cells and reagents

Human PaCa cell lines (L3.6pl, BxPC-3, Mpanc96, Panc-1, and Miapaca-2) were provided kindly by Dr. C. Logsdon (Department of Cancer Biology) and Dr. S. Guha (Department of Gastroenterology,Hepatology&Nutrition) at U. T. MD Anderson Cancer Center and have been described previously [23], [31]. All cell line identities were verified by DNA fingerprinting. Cells were cultured in RPMI 1640 supplemented with 10% fetal bovine serum (FBS) and antibiotics (100 µg/mL streptomycin and 100 IU/mL of penicillin). Anti-Gal-3 antibody was obtained as described previously [8]. Anti-Ras monoclonal antibody, anti-RalA monoclonal antibody, anti-phospho-AKT and anti-phospho-ERK antibodies were from Cell Signaling Technology, Inc. (Beverly, MA). Anti-cyclin D1 and anti- C-MYC antibody was from Santa Cruz Biotechnology, Inc. (Santa Cruz, CA).

### Protein isolation and immunoblot analysis

Total pancreatic cancer cell lysates including human and a K-Ras G12D mouse model [25] were prepared in 2% SDS lysis buffer as previously described [9]. Cytoplasmic and nuclear extractions were prepared using NE-PER Nuclear and Cytoplasmic Extraction Reagents (Pierce Biotechnology, Rockford, IL) according to the manufacturer's instructions. Western blot analyses were performed as previously described [9].

### Generation of stable Gal-3 silencing and overexpressing PADC cell lines with lentiviral shRNA

To produce lentivirus for Gal-3 overexpression (L-gal3) or Gal-3-shRNA (A3), pLVTHM-Gal3 or pLVTHM-shGal3, respectively, and control vector were cotransfected with the packaging plasmid (MD2G) and envelope plasmid (PAX2) required for viral production into 293FT cells by using lipofectamine 2000 reagent (Invitrogen). Medium containing lentivirus with Gal-3 shRNA(named A3) and control vector (named GN10) was used to transduce Mpanc96, Miapaca-2 and Panc-1 cell lines with high levels of Gal-3 expression. Medium containing lentivirus with Gal-3 overexpression (named L-gal-3) and control vector (named V) was used to transduce BXPC-3 and L3.6pl PaCa cell lines with low Gal-3 expression. Cell sorting was conducted in a fluorescence-activated cell sorting ARIA flow cytometer (BD Biosciences). Gal-3 expression of stable cell lines was further confirmed by Western bloting.

### Cell proliferation assay

Cell viability was measured using the CellTiter Aqueous One Solution Cell Proliferation Assay kit as described [24].

### Soft agar colony formation assay

5×10^3^ MPanc96 cells stably expressing pLVTHM-shRNAGal3 or pLVTHM control vector were mixed with 0.6% agar in DMEM. The cell/agar mixtures were placed in 6-well plates coated with 0.3% agar in triplicate. The cells were incubated at 37°C for 10 days. Colonies were stained with Diff-Quik (Dade Behring). The experiment was repeated three times independently.

### Co-immunoprecipitation

Co-immunoprecipitation in control GN10 and Gal-3 shRNA A3 cells from both Panc-1 and Mpanc96 cells were performed as described [9].

### Ras and Ral A activity assay

Equal amounts of cell lysate protein from GN10 control cells or A3 shRNA gal-3 cells from Panc-1 and Mpanc96 or BXPC-3 L-gal-3 and its control BXPC-3 V were incubated for 45 min at 4°C, with agarose beads coated with Raf1-RBD for total Ras activity or agarose beads coated with Ral BP1 agarose for total Ral A activity (Upstate Biotechnology, Inc., Lake Placid, NY). Beads were then washed and bound protein was eluted with 2× Laemmli sample buffer that had been preheated to 95°C and analyzed by immunoblotting for Ras using anti-Ras antibody or RalA activity using anti-RalA antibody.

### Matrigel invasion assay

The invasive capability of cells was determined by using Matrigel-coated invasion chambers with 0.8-µm pore size (BD Biosciences). A single-cell suspension containing 1×10^5^ cells was added to the inner chamber. After 16 h of incubation at 37C in 5% CO2, cells on the upper surface of the inner chamber were removed with cotton swabs. Invaded cells that adhered on the lower surface of the membrane were fixed, stained with Diff-Quik (Dade Behring), and counted.

### Indirect immunofluorescence staining and confocal laser microscopy

Indirect immunofluorescence staining in BXPC-3 cells were performed as described [9]. Expression and localization of the proteins were observed under a confocal microscope system (FluoView FV500; Olympus, Melville, NY) and analyzed by CellQuest PRO software (BD Biosciences, San Jose, CA) at the Flow Cytometry and Image Analysis Core Laboratory at The University of Texas M. D. Anderson Cancer Center.

### Real-time polymerase chain reaction

To quantify the changes in Gal-3 mRNA levels, real-time RT-PCR was performed on the ABI Prism 7900 (Applied Biosystems, Foster City, CA) using the commercially available gene expression assay for Gal-3 (Mm00802901_m1), and the cyclophilin A (4326316E; Applied Biosystems) as described previously [9]. The 7900 Sequence Detection System 2.2 software (Applied Biosystems, Foster City, CA) automatically determined the fold-change for Gal-3 in each sample by using the δδCt method with 95% confidence.

### Immunohistochemistry

Immunohistochemical staining for Gal3 was performed on microarray tissue slides consisting of 125 pancreatic ductal adenocarcinomas and their paired non-neoplastic pancreatic tissues from patients who underwent pancreaticoduodenetomy at MD Anderson Cancer Center. This study was approved by the Institutional Review Board of M. D. Anderson Cancer Center. After antigen retrieval and endogenous peroxidase blockage, the sections were then incubated with antibody against Gal3 (1∶100 dilution) at 4°C overnight, then incubated with secondary antibody at room temperature for 60 min. Standard avidin-biotin immunohistochemical analysis of the sections was performed according to the manufacturer's recommendations (Vector Laboratories, Burlingame, CA). The staining results were evaluated by a pathologist (H.W.) based on the percentage of staining in tumor cells (0, no staining; 1, ≤10%; 2, 10–50% and 3, >50%) and the staining intensity (0-negative, 1-weak, 2-moderate and 3- strong). The tumors were categorized into Gal3-low (combined scores≤3) and Gal3-high (combined score ≥4).

### Othotopic PADC model

MPanc96 cells stably transfected with Gal-3 shRNA and corresponding control vector were stably transduced with luciferase as previously described [25], [26]. Animals were divided into 2 groups (n = 5 per group). The first group was injected with MPanc96 cells that were transfected with vector (GN10) only and the second group with MPanc96 cells transfected with Gal-3 shRNA (A3). Animals were anesthetized with ketamine-xylazine solution, a small left abdominal flank incision was made, and (1×10^6^) MPanc-96 cells in 50 µL PBS were injected into the subcapsular region of the pancreas using a 27-gauge needle and a calibrated push button–controlled dispensing device (Hamilton Syringe Company). The abdominal wound was closed in one layer with wound clips (Braintree Scientific, Inc.).

One week after implantation, tumor volumes were monitored weekly by non-invasive real-time bioluminescence imaging by IVIS 200 (Xenogen) using a cryogenically cooled bioluminescence imaging system coupled to a data acquisition computer running Living Image software (Xenogen) as previously described [23]. Mice were imaged on day 7, 14, 21, and 28 after tumor cells implantation. The animals were sacrificed on 28th day after tumor cell implantation. Primary tumors in the pancreas were excised, and the final weight was measured. All the measurements were compared among two groups using unpaired Student's *t* test. The tumor tissue and surrounding organs were embedded in paraffin and serial 5-µm sections were cut, stained with hematoxylin-eosin, and examined by light microscopy to verify the presence of tumor and microscopic metastases. Tumor tissues were processed for immunohistochemistry and stained for expression of galectin-3, Ras and phopho-ERK. as described above.

### Statistical analysis

Assays are presented in graphs as mean ± SD and represent the results of at least three experiments. Significance of differences between the groups was judged using a two-tailed Student *t* test. Results were considered statistically significant if the *P* value was <0.05.

## Results

### Gal-3 is highly up-regulated in human pancreatic tumor tissues

**Figure 1:**
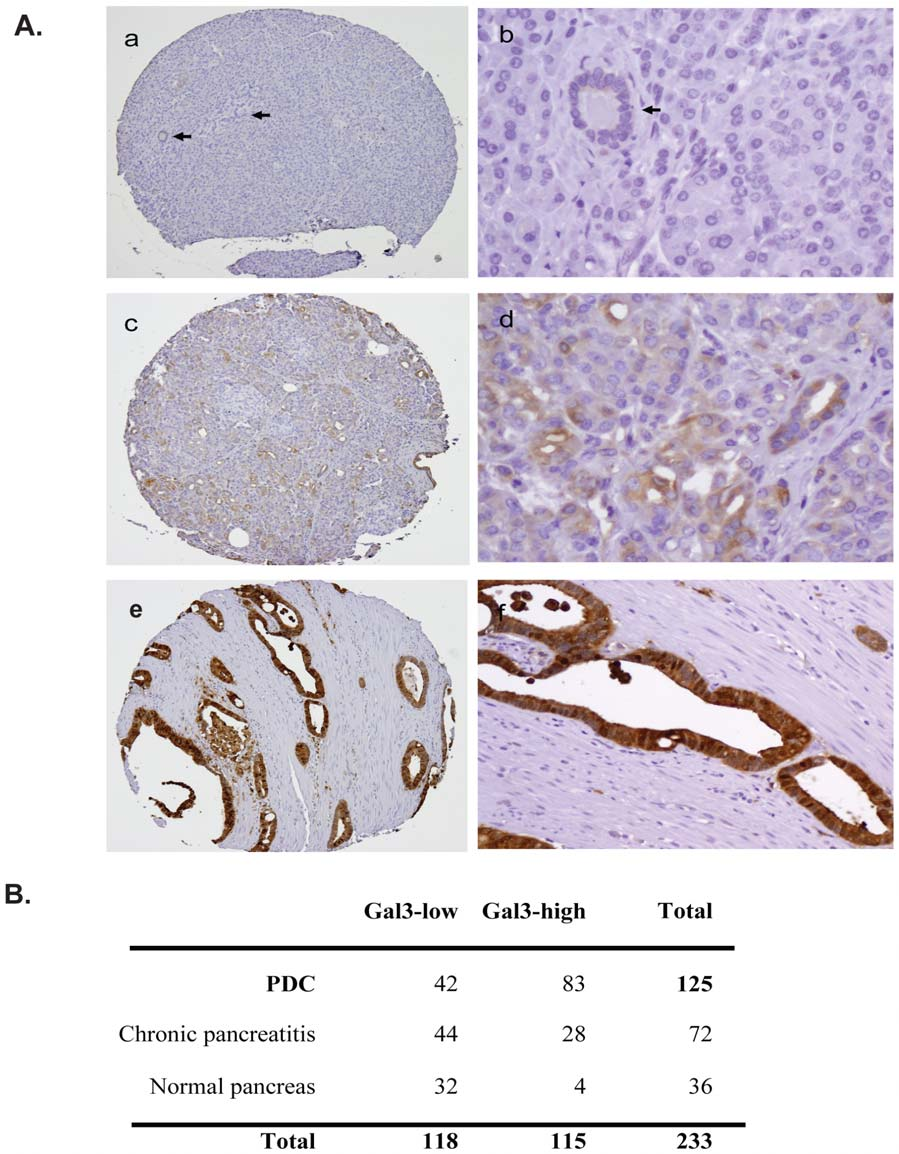
Gal-3 expression in human pancreatic tumor tissues. A, Tissue microarray slides consisting of 125 pancreatic ductal adenocarcinomas and paired non-neoplastic pancreatic tissue samples were immunohistochemically stained using a monoclonal Gal-3 antibody as described in Materials and Methods. Gal-3 expression was increased along the disease sequence-normal (Figure 1A, a,b), pancreatitis (c,d) and pancreatic ductal adenocarcinoma (e,f). B. Summary of Gal-3 IHC of the PDAC tissue microarrays. The tumors were categorized into Gal3-low (combined scores ≤3) and Gal3-high (combined score ≥4) based on the percentage of Gal-3 staining in tumor cells (0, no staining; 1, ≤10%; 2, 10–50% and 3, >50%) and the staining intensity (0-negative, 1-weak, 2-moderate and 3- strong).

Gene expression profiling studies suggest that Gal-3 is up-regulated in pancreatic tumors compared to control tissues [18], [19]. We performed immunohistochemical staining of human pancreatic tissue microarrays with anti-Gal3 antibody (Figure 1A). We found that Gal-3 expression progressively increased in the sequence of disease progression normal (Figure 1A, a, b), pancreatitis (c,d) and pancreatic ductal adenocarcinoma (e,f). Gal-3 staining was categorized into two groups, Gal-3 Low (score <3) and Gal-3-high (score >4), based on the staining intensity and positive staining percentage as described in Materials and Methods. We found that 83 of 125 (66.4%) pancreatic ductal adenocarcinoma samples showed high levels of Gal-3 expression (Gal3-high). Among these cases, paired non-neoplastic pancreatic tissues were available for evaluation in 108 cases (72 samples of chronic pancreatitis and 36 normal pancreatic tissue samples) and 32 (29.6%) were Gal3-high. Gal-3 expression was significantly higher in pancreatic ductal adenocarcinoma than the paired non-neoplastic pancreatic tissue samples (p<0.0001). 28 of 72 (38.9%) chronic pancreatitis samples were Gal3-high compared to 11.1% (4/36) of normal pancreatic tissue samples (P<0.001) (Figure 1B). These data indicate that Gal-3 is up-regulated during the human pancreatic disease progression normal, pancreatitis and pancreatic ductal adenocarcinoma.

### Gal-3 is highly up-regulated in pancreatic tumor tissues and cells from a K-Ras mutant mouse model

**Figure 2:**
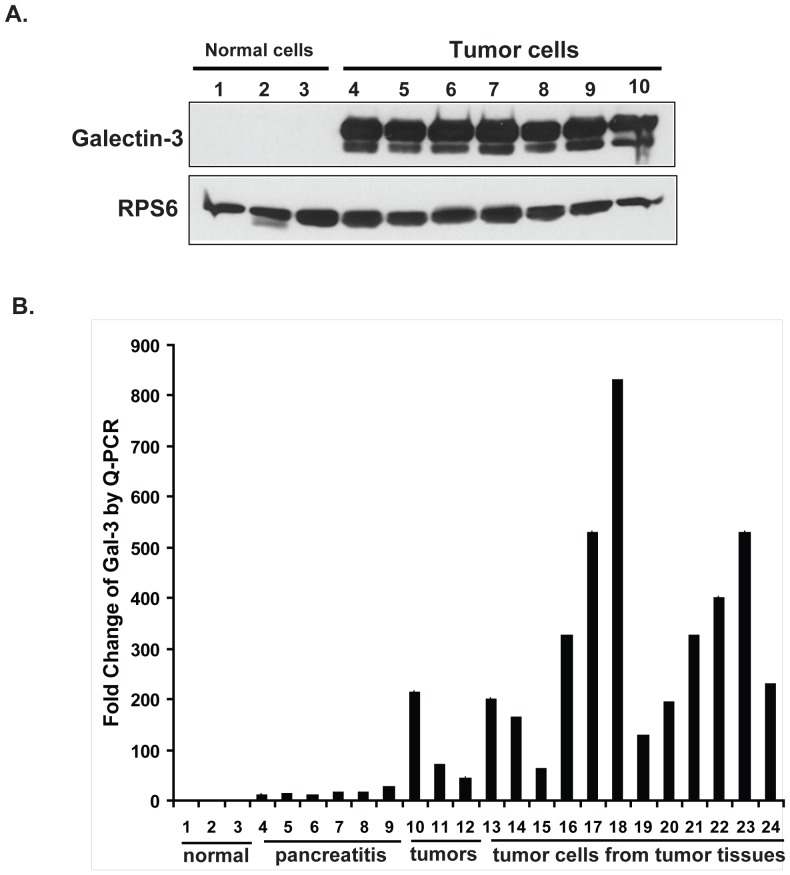
Gal-3 expression in pancreatic tumor tissues and cells from a K-RAS mutant mouse model. A. Gal-3 expression was analyzed in cells derived from normal pancreas and pancreatic tumors from K-Ras mutant mice by western blotting as described in Materials and Methods. B. Real-time quantitative PCR for Gal-3 RNA expression using Gal-3-specific primers after total RNA extraction from normal pancreas, pancreatitis tissues and pancreatic tumors and from isolated cells from different tumors arising in a K-Ras mutant mouse model as described in Materials and Methods. Fold change from determinations performed in triplicate.

A recently described mutant K-Ras mouse model recapitulates the human pancreatic cancer progression sequence from inflammation to PanIN to PDAC [25]. It was of interest to determine whether this mouse model would also mimic the changes in Gal-3 expression observed in human samples. As shown in Figure 2A, Gal-3 was undetectable in pancreas tissues from normal mice (lanes 1–3), but highly up-regulated in seven tumor cell lines (lanes 4–10) which were derived from seven different mouse tumors.

In order to determine if Gal-3 mRNA was also up-regulated in mouse tumor tissues compared with normal and pancreatitis tissues, tissues from individual mice that were normal, had pancreatitis or those with tumors were extracted and real-time PCR was performed using specific mouse Gal-3 primers (Mm00802901_m1, Ambion, USA) (Figure 2B). We observed that Gal-3 expression was low in normal pancreatic tissues (lanes 1–3), but was increased 10–27 fold in chronic pancreatitis (lanes 4–9) and increased 45–215 fold in K-RAS mutant mouse tumor tissues (lanes 10–12). Gal-3 mRNA levels were even higher (62 fold to 831 fold), when measured in isolated tumor cells (lanes 13–24).

### Generation of Gal-3 over- and under-expressing PDAC Cells

**Figure 3:**
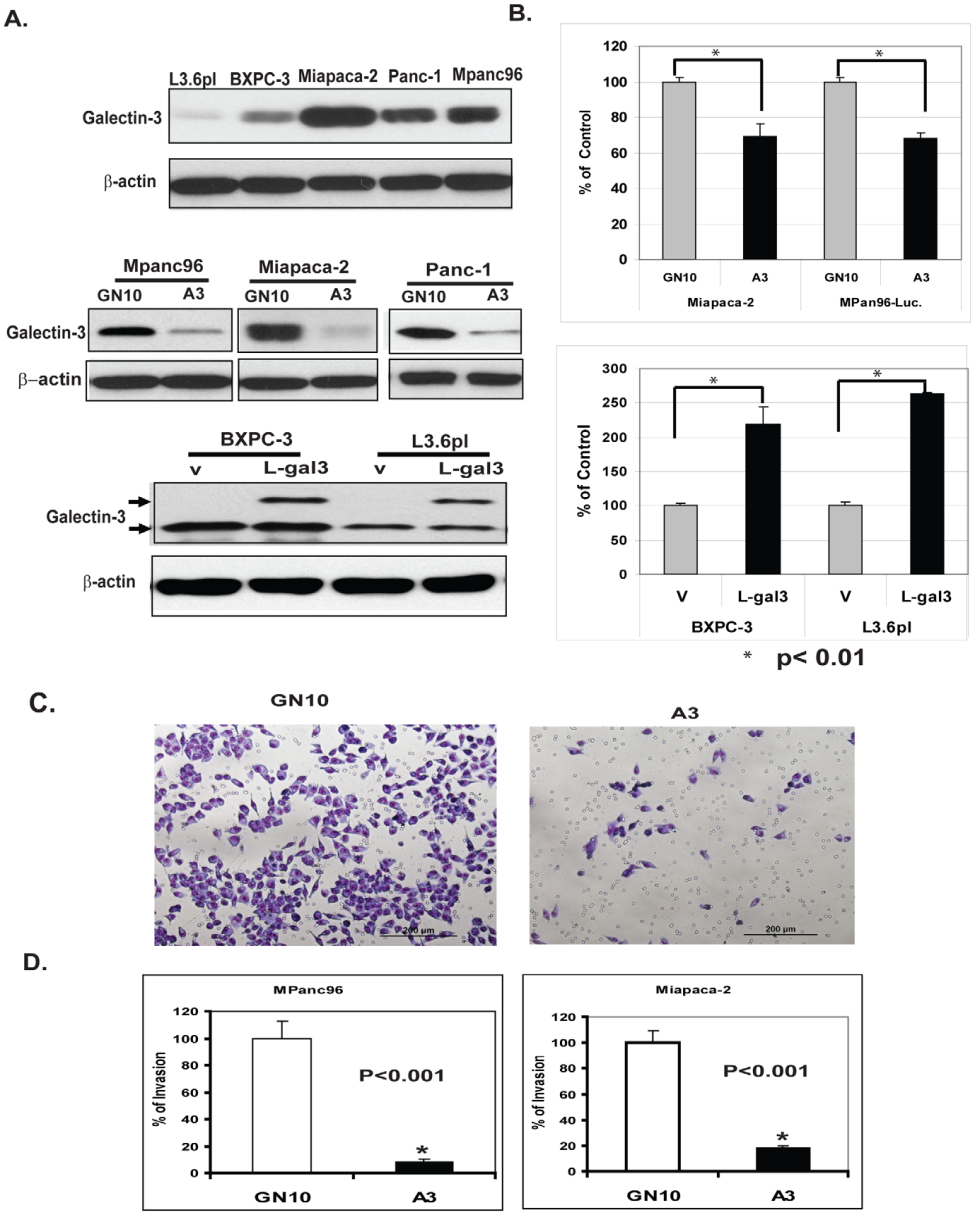
Validation of Gal-3 expression in stably transformed PDAC cells and effects of Gal-3 on cell proliferation and invasion. A. Gal-3 expression in different PDAC cell lines as determined by Western blotting (top panel). Knockdown Gal-3 by lentivirus shRNA of Gal3 (A3) or control vector (GN10) in Gal-3 high cell lines- Mpanc96, Miapaca-2 and Panc-1 were determined by immunoblots (Figure 3A, middle panel). Overexpression Gal-3 in BXPC-3 and L3.6pl cells lower Gal3 expressed cells by lentivirus infection of Gal3 cDNA (L-gal3) or vector control (v) was confirmed by immunoblots (Figure 3A, lower panel. B. Effects of Gal-3 on cell proliferation. Proliferation of Miapaca-2,and Mpanc96 cells in which Gal-3 was down-regulated by shRNA or BXPC-3 and L3.6pl cells in which over-expressed by Gal-3 cDNA was determined by using the CellTiter Aqueous One Solution Cell Proliferation Assay kit as described in Materials and Methods. C. Effect of Gal-3 on cancer cell invasion in PDAC cells. Representative fields are shown PDAC cells (1×10^5^) in which Gal-3 expression was knocked down by shRNA (A3) and vector transfected control cells (GN10) were seeded onto Matrigel-coated invasion chambers; 24 hours later, invaded cells that adhered on the lower surface of the membrane were fixed, stained with Diff Quick set, and count as described in Materials and Methods. D. Bar graph for percent of Invasion in both Mpanc96 and Miapaca-2 cells with Gal3 knockdown (A3) or Control (GN10) represents the mean of three independent experiments performed in triplicate; bars, standard errors, p<0.001.

In order to manipulate Gal-3 levels in PADC cells, we first determined the level of Gal-3 expression in a variety of PADC cell lines (Figure 3A, top panel). MPanc96, MIAPaCa-2 and Panc-1 cells had high basal Gal-3 expression (Figure 3A) and were stably transfected with lentivirus Gal-3 shRNA (A3 cells) or vector only (GN10 cells). Galectin-3 levels were successfully reduced in Miapaca-2, Panc-1 and Mpan96 cells 90%, 95% and 92% respectively (Figure 3A, middle panel). In order to study the effects of up-regulation of galectin-3, Gal-3 cDNA lentivirus were transfected into PDAC cells with lower basal Gal-3 levels (Figure 3A, lower panel). These variants were designated as L-gal3 and V (vector control) as shown in Figure 3A, lower panel. These PDAC cells were used to further determine the function of Gal-3 in the pathogenesis of pancreatic cancer.

### Gal-3 regulates cell proliferation, invasion and colony formation in PADC cells in vitro

In order to determine if Gal-3 plays a functional role in pancreatic tumor cell behavior in vitro, we utilized cell lines genetically altered to express different Gal-3 levels as shown in Figure 3A to determine the effects of Gal-3 on tumor cell growth (MTS assay), invasion (Matrigel invasion assay) and in vitro tumorigenicity (soft agar colony formation assay). Knock down of Gal-3 in Miapaca-2 and Mpanc96 cells significantly decreased cell growth at 72 hours (Figure 3B, top panel). In a complementary experiment, over-expression of Gal-3 in L3.6PL and BXPC-3 cells (L3.6PL-L-gal3 and BXPC-3-L-gal3) increased cell proliferation compared with control cells L3.6PL-V and BXPC-3-V (Figure 3B, Lower panel). To investigate whether Gal-3 affects the invasive capabilities of PDAC cells, Matrigel invasion assays were performed. Downregulation of Gal-3 (A3) in MPanc96 and Miapaca-2 cells decreased cell invasion by 95% and 90% respectively compared with control cells (Figure 3C and 3D). In contrast, Gal3 cDNA expressed cells BXPC-3 L-gal3 increased cell invasion capacity (data not shown). These results indicate that Gal-3 also plays an important role in the invasion of PDC cells.

**Figure 4:**
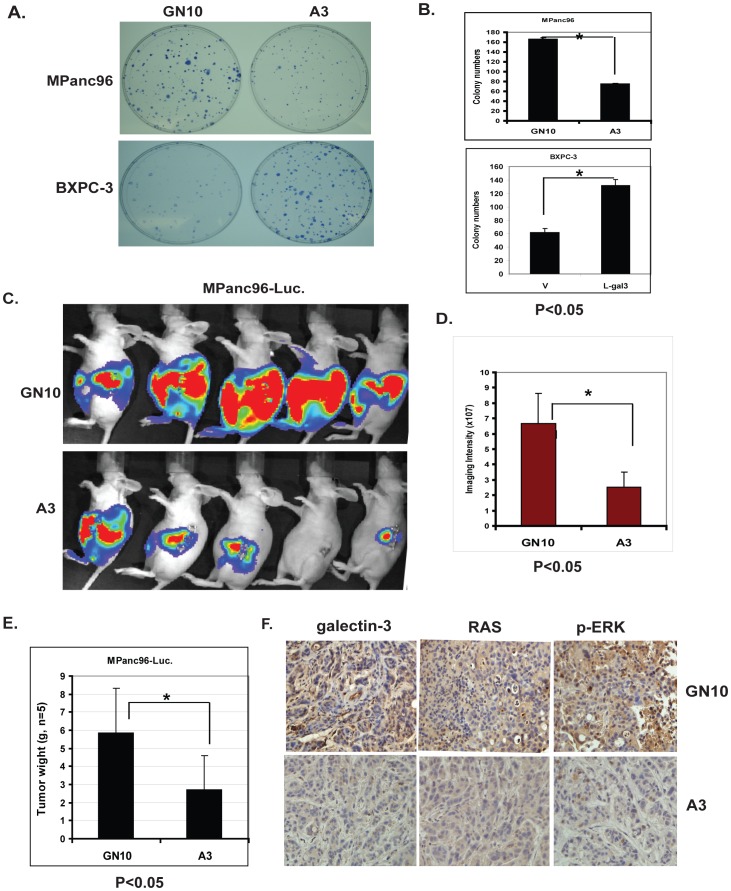
Effects of Gal-3 on PDAC cell colony formation and the growth of PDAC tumor in vivo. A. Colony formation assays were performed in MPanc96 GN10 control cells and Gal-3 shRNA knock down A3 cells (top panel) or BXPC-3 V control cells and BXPC-3 Gal3 cDNA overexpressed L-gal3 cells (lower panel) as described in Materials and Methods. MPanc96-A3 cells formed smaller and fewer colonies compared with the vector transfected control cells (GN10). In contrast, BXPC-3 L-gal3 cells formed larger and a greater number of colonies than control V cells. B. Bar graph in the right panel demonstrates the mean colony numbers after plating either MPanc96 GN10 control cells and shRNA knock down A3 cells (top) or BXPC-3 V and BXPC-3 L-gal3 (lower); p<0.001. C. Representative bioluminescence images of athymic mice 3 weeks after orthotopic implantation of GN10 and A3 pancreatic cancer cells orthotopically into the pancreas of athymic mice. D. Measurements of photons/s/cm^2^/steridian depicting bioluminescence area at 10% peak margin (mean ± SE) at week 3 using Xenogen IVIS as described in Materials and Methods (*n* = *5*). E. Tumor weights from control mice (GN10, n = 5) and Gal3 knockdown group (A3, n = 5) were weighted after mice were sacrifice at week four. F. Mouse tumor tissues from control (GN10) and Gal-3 knockdown group (A3) were immunohistochemically stained using Gal-3, Ras and phospho-ERK antibodies as described in Materials and Methods.

In addition, down-regulation of Gal-3 by shRNA in MPanc96 cells (MPanc96 A3) dramatically decreased colony numbers in soft agar compared with vector-transfected controls (Figure 4A, 4B top panels), while up-regulation of Gal-3 in BXPC-3 cells significantly increased colony numbers compared with controls (Figure 4A,4B lower panels). These data indicate that Gal-3 levels influence PDAC cell proliferation, invasion and anchorage-independent growth.

### Downregulation of Gal-3 inhibits tumor growth in an in vivo orthotopic model

To investigate the influence of Gal-3 levels on in vivo growth of PDAC, MPanc96 cells stably transfected with Gal-3 shRNA or a control shRNA vector were labeled with firefly luciferase to allow real-time bioluminescence imaging to monitor tumor volume and spread in vivo. These cells were orthotopically injected into the body of the pancreas of nude mice, and tumor growth was monitored by non-invasive imaging once per week. Differences in the growth of the tumors were noted within 3 weeks (representative images as shown in Figure 4C). Quantitative evaluation of tumor burden indicated that tumors were significantly larger in the control group compared to the Gal-3 knock down group (Figure 4D) (p<0.05). In addition, primary tumor weights were significantly less in the Gal3 knock down group than the control group as demonstrated in Figure 4 E; Immunohistochemical staining for Gal-3, Ras and phospho-ERK in these mice tumor tissues (Figure 4F) further confirmed that expression of Gal-3, Ras and the down-stream effector phospho-ERK are decreased in the Gal-3 knock down group compared with the control group. Regional micrometastases were noted in animals injected with control cells (5/5 animals), but not in animals injected with cells in which galectin-3 had been knocked down by stable transfection of galectin-3 shRNA (0/5 animals) (Figure S1).

### Gal-3 binds to Ras and increases Ras activity in PDAC cells

**Figure 5:**
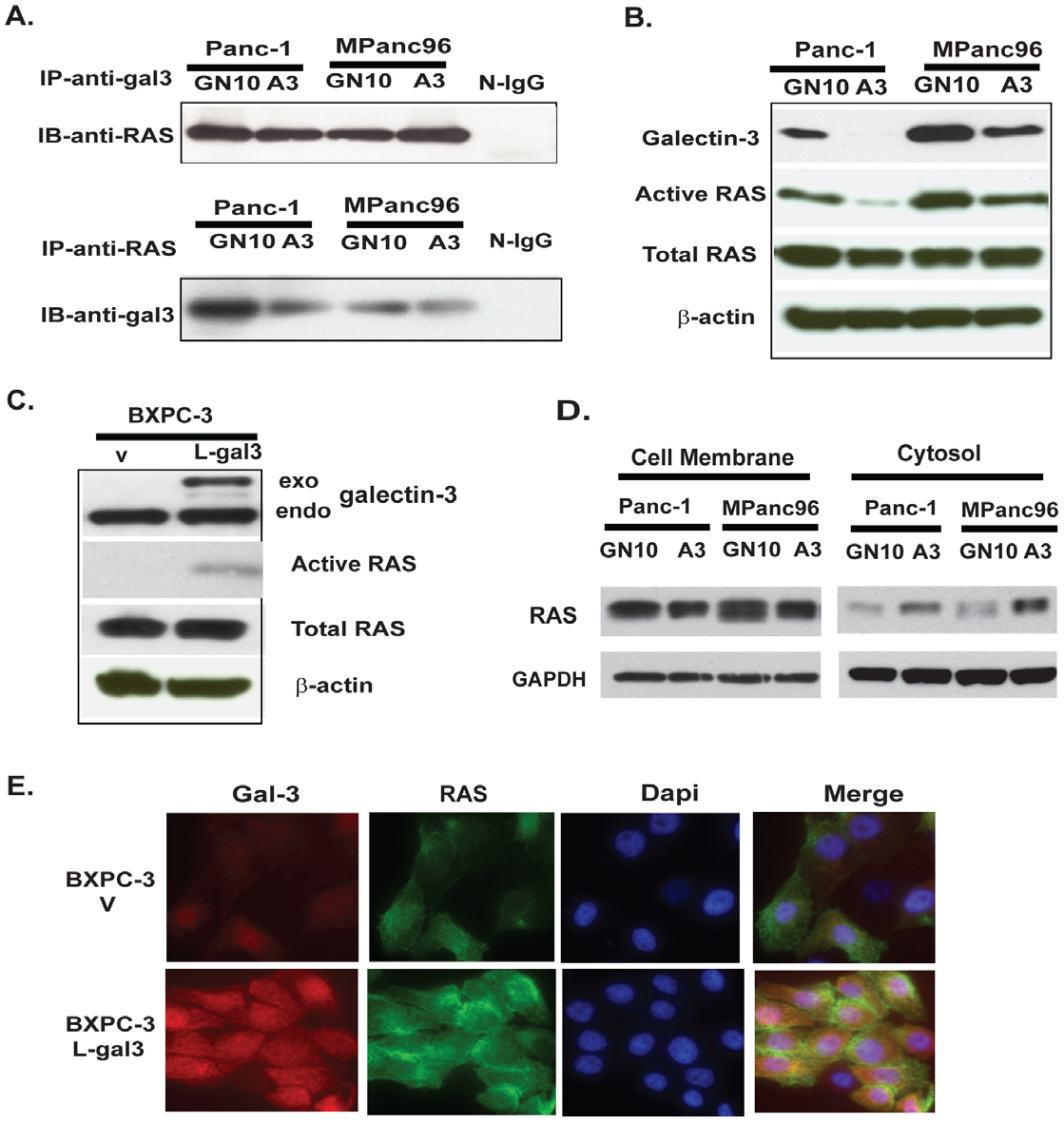
Gal-3 binds to Ras and mediates Ras activity in PDAC cells. A. Co-Immunoprecipitation was performed in Panc-1 and MPanc96 with knock down Gal-3 using either anti-Ras or anti-Gal-3 antibody as described in Materials and Methods. B. Equal amounts of protein from Panc-1 and Mpanc96 GN10 control cells or shRNA A3 cells were incubated with agarose beads coated with Raf1-RBD, and active Ras-GTP was detected as described in Materials and Methods. C. Active Ras GTP activity was determined in BXPC-3 L-gal3 cells and control cells (V) as described in Materials and Methods. D. Cell plasma membrane and Cytoplasmic fractions of GN10 and A3 cells from both Panc-1 and MPan96 were subjected to SDS-PAGE and then immunoblotted with anti-Ras antibody. E. Indirect immunofluorescence was performed on BXPC-3-V and BXPC-3 L-gal3 cells using anti-Gal-3 antibody (TIB166 1∶100, red) and anti-Ras antibody (1∶100, green), followed by DAPI counterstaining (blue). The merge of Gal-3 (red) and Ras (green) with DAPI (blue) is also shown.

It has previously been reported that Gal-3 is associated with activated K-Ras and promotes strong activation of Raf-1 and PI3-K but attenuates activation of ERK signaling in COS cells [21]. We therefore hypothesized that Gal-3 would bind with Ras and mediate Ras activity in PDAC cells characterized by the frequent occurrence of Ras mutations. Using an antibody array (Hypromatrix, Worcester, MA), we detected the interaction between Ras and Gal-3 using HA tagged Gal3 transfected BXPC-3 lysate (data not shown). Ras was confirmed to be one of the binding partners of Gal-3 based on data from the antibody array. To further confirm that Ras interacts with Gal-3, we used co-immunoprocipitation assays. Lysates from Panc-1 and MPanc96 with or without knock down of Gal-3 were subjected to immunoprecipitation with either Gal3 rat monoclonal antibody or a pan anti-Ras monoclonal antibody followed by immunoblotting with anti-Ras or ant-Gal3 antibodies. Results demonstrated that Gal-3 co-immunoprecipitated with Ras, while lysates with normal IgG yielded no visible band (Figure 5A).

To further determine if interaction between Ras and Gal-3 affects Ras activity, we examined the effects of manipulating Gal-3 expression on Ras activity. As shown in Figure 5B, down-regulation of Gal-3 in Panc-1 and MPan96 cells (A3) decreased Ras activity using the Ras-GTP assay, while overexpression of Gal3 in BXPC-3 cells increased Ras GTP activity (Figure 5C). In order to further elucidate how Gal-3 mediates Ras activity, cell membrane and cytosolic proteins were isolated [26] from Panc-1 and MPanc96 control cell (GN10) and knock down cells (A3) and Ras protein was detected using immunoblotting. Less Ras protein was detected in the plasma membranes of Panc-1 A3 cells and Mpanc96 A3 cells compared to control GN10 cells (Figure 5D). In contract, more RAS protein was observed in the cytosol of Panc-1 and Mpanc96 A3 cells compared to control GN10 cells. These data suggest that Gal-3 may govern Ras subcellular localization. Confocal immunofluorescence confirmed that up-regulation of Gal-3 in BXPC-3 cells is associated with increased Ras at the plasma membrane (Figure 5E).This suggests that Gal-3 binds Ras and is responsible for Ras attachment to the cell plasma membrane, thereby mediating its activity.

### Gal-3 mediates Ras downstream signaling in PDAC cells

**Figure 6:**
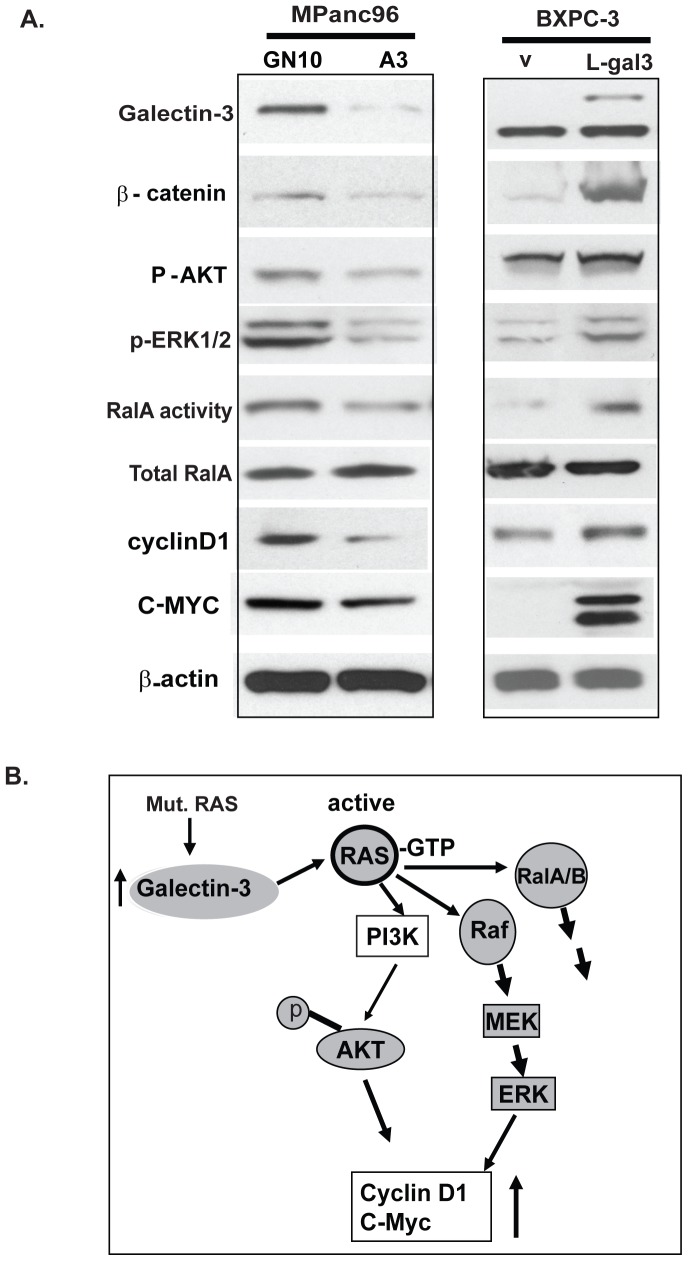
Gal-3 mediates Ras downstream signaling in PDAC cells. A. Total cell lysates of paired MPanc96 GN10/MPanc96 A3 cells and BXPC-3 V/BXPC-3 L-gal3 cells were prepared and immunoblots were performed for Gal-3, phospho-AKT, ERK1/2, β-catenin, cyclinD1, C-MYC, total Ral A. Ral A activity was performed as described in Materials and Methods. B. Proposed model by which Gal-3 mediates Ras signaling in PDAC cells. Up-regulated Gal-3 in pancreatic tumor cells, feeds forward to enhance Ras oncogenic signaling by binding to Ras and augment its downstream signaling, thereby mediate tumor cell proliferation and invasion.

Activated Ras (GTP-bound state) activates downstream effectors including the Raf/mitogen-activated protein (MAP)/extracellular signal-regulated kinase (MEK/ERK), phosphatidylinositol-3-OH kinase/AKT, and RalA pathways to induce a range of cellular responses including proliferation, migration and transformation. We therefore wished to determine the effects of Gal-3 on Ras down-stream signaling. Downregulation of Gal-3 in MPanc96 pancreatic cancer cells was associated with decreased phosphorylation of AKT, ERK1/2 and also RalA-GTP activity (Figure 6A, left panel). Silencing Gal-3 also reduced known Ras target genes including β-catenin, cyclin D1 and C-MYC (Figure 6A), In contrast, up-regulation of Gal-3 in BXPC-3 cells with low basal Gal-3 expression increased phosphorylation of AKT, ERK1/2, and RalA activity as well as the expression of β-catenin, cyclinD1 and C-MYC (right panel). This suggests that down-regulation of Gal-3 decreases Ras down-stream signalings (left panel); while up-regulation of Gal-3 increases Ras down-stream signaling (right panel).

## Discussion

Galectin-3 (Gal-3), a member of the animal lectin family, is up-regulated in many tumors including pancreatic cancer [7], [14], [18]. Its importance in pancreatic cancer, a tumor characterized by frequent Ras mutation, however, remains unknown. In this study, we demonstrate that Gal-3 in pancreatic cancer tissue and cells, whether mouse or human, is highly elevated. Our data suggests that elevated Gal-3 in pancreatic tumor cells with mutant K-Ras further increases Ras activity by binding Ras to the plasma membrane, where mediates its downstream effectors including Raf/MEK/ERK,PI3K/AKT and RalA (Figure 6B), thereby inducing pancreatic cancer cell growth and invasion in vitro and in vivo. Gal-3 expression has been previously reported to be elevated in human pancreatic tumors compared with normal tissues, although the clinical significance of this finding has been unclear [12], [15], [18]. In the current study, we confirm that expression of Gal-3 is elevated in human pancreatic tumor tissues in a larger tissue microarray set, as well as in a mouse model of pancreatic tumorigenesis which mimics the human condition [25]. In this model, mice with mutations in the K-Ras gene develop chronic pancreatitis, acinar to ductal metaplasia and a high incidence of PDAC at a relative early age[3], [25]. Our findings demonstrate that mutant K-Ras is associated with high Gal-3 expression in this model, This is in agreement with previous findings that Gal-3 expression increased in Ras transformed NIH3T3 cells, human HOS cells [27] and in p53 mutant cells [28].

We have demonstrated that down-regulation of Gal-3 by ShRNA in several pancreatic cancer cell lines with high constitutive Gal-3 expression decreases cell proliferation and invasion in vitro and tumor volume and size in vivo in an orthotopic pancreatic cancer mouse model. This finding further suggests that elevated Gal-3 in tumor cells plays an important functional role in tumor cell growth and invasion. A recent publication by Kobayashi et al [16] reported that transient gene silencing of galectin-3 suppresses pancreatic cancer cell migration and invasion, but failed to affect proliferation. The discrepancy between our findings and those reported by these authors could be due to different degrees of galectin-3 inhibition. In our study lentivirus-shRNA was used to generate stable clones in which the efficiency of knock down is robust and stable, while Kobayashi et al employed transient silencing of galectin-3. Hann et. al. [29] studied a variety of pancreatic cell lines in vitro and in vivo (subcutaneous xenografts) after manipulation of galectin-3 levels and concluded that galectin-3 had no consistant oncogenic function in pancreatic cancer cells. While this group did not note a universal effect on in vitro characteristics such as proliferation, migration or anchorage independent growth after transient tranfection of galectin-3 si-RNA in all cells lines, there was a moderate effect in several cell lines. Both our report and that of Kobayashi et. al. [16] provide clear evidence for an effect of galectin-3 on cell migration and invasion, properties associated with tumor progression. Lack of effect of galectin-3 knockdown on growth of subcutaneous xenografts is interepreted by these authors as evidence for a lack of effect of galectin-3 on oncogenic function. Using orthotopic implantation into the pancreatic bed, a model which better recapitulates in vivo pancreatic tumor growth, we have clearly demonstrated an effect of galectin-3 on pancreatic tumor growth.

The molecular mechanisms by which Gal-3 mediates cell proliferation and invasion in pancreatic cancer are unclear. Studies in human breast cancer cells [30] have demonstrated that Gal-3 mediates K-Ras GTP nanocluster formation and its signal output and the transformed phenotype of breast cancer cells [30]. It has been recently demonstrated that K-Ras GTP in association with Gal-3 contributes to the development of thyroid carcinoma, and that the Ras inhibitor FTS disrupts the interactions between K-Ras and Gal-3 and inhibits thyroid tumor growth [22]. Since mutations in K-Ras are nearly universal in PDAC and Gal-3 is highly expressed in pancreatic cancer cells in both human and K-Ras mutant mouse, we asked whether elevated Gal-3 levels may further mediate or augment Ras activity in pancreatic cancer cells. Our results suggest that Gal-3 levels modulate Ras activity in pancreatic cancer cells, since down-regulation of Gal-3 decreases Ras activity while up-regulation of Gal-3 increases Ras activity in pancreatic cancer cells. Using antibody array based screens and immunoprecipitation assays, we found that Gal-3 can bind to Ras in pancreatic cancer cells and retain Ras in the plasma membrane where it activates its downstream signaling. These findings are consistant with previous findings in which breast cancer cells were studied [31]. Kloog and colleagues have studied the role of galectins by manipulation of cultured NIH3T3 cells, embryonic fibrobasts, breast cancer cell lines and thyroid cancer cell lines [22], [30]–[32]. Recent study from Levy R at al demonstrated galectin-3 mediates cross-talk between K-Ras and Let-7c tumor suppressor miRNA [33]. These studies for the most part examine the effects of galectin-3 on signaling pathways in cultured cells, or infer an effect on oncogenic function using in vitro assays such as anchorage independence or resistance to apoptosis, or in the case of thyroid cancer subcutaneous growth. We believe that our use of complementary in vitro and in vivo systems (including a mutant K-ras mouse mode and orthotopic implantation) with a special emphasis on pancreatic carcinogenesis (where abnormal K-ras is clearly functionally important) adds important information to the existing literature.

Ras mutations are early and prevalent genetic events in pancreatic cancer development. However, K-Ras mutations have also been observed in 30–40% of samples from patients with CP and may even be present in normal individuals [5]. Low levels of mutant K-Ras activity may not be sufficient to meet a threshold of pathologic Ras signaling necessary for tumorigenesis. Based on our results it is possible that up-regulation of Gal-3 may feed forward to promote Ras oncogenic signaling by binding Ras and augmenting its downstream signaling (Figure 6B).

In conclusion, we have demonstrated that Gal-3 is highly up-regulated in pancreatic tumor tissues and cells in both human pancreas and in a K-Ras mutant mouse model of pancreatic cancer. Our data suggest that up-regulated Gal-3 in tumor tissues further enhances Ras activity by binding and retaining Ras at the plasma membrane, thereby activating down-stream Ras signaling. Gal-3 may therefore play a pivotal role in the pathogenesis of pancreatic cancer, in which Ras mutations frequently occur. Targeting Gal-3 could therefore be important therapeutic modality for this deadly disease.

## Supporting Information

Figure S1 **Micrometastases (indicated with arrows) in MPanc96 GN10 mice but not in A3 mice.** Representative tumor tissues were stained HE (Upper panel) and Gal-3 IHC (Lower panel) from Mpanc96 Control mice (GN10) and Gal-3 knock down (A3) mice.
(TIF)
