# Supplementary figures and images for: Overexpressed Galectin-3 in Pancreatic Cancer Induces Cell Proliferation and Invasion by Binding Ras and Activating Ras Signaling
Source: PLoS One. 2012 Aug 10;7(8):e42699. doi: 10.1371/journal.pone.0042699 (PMC3416861; doi:10.1371/journal.pone.0042699)

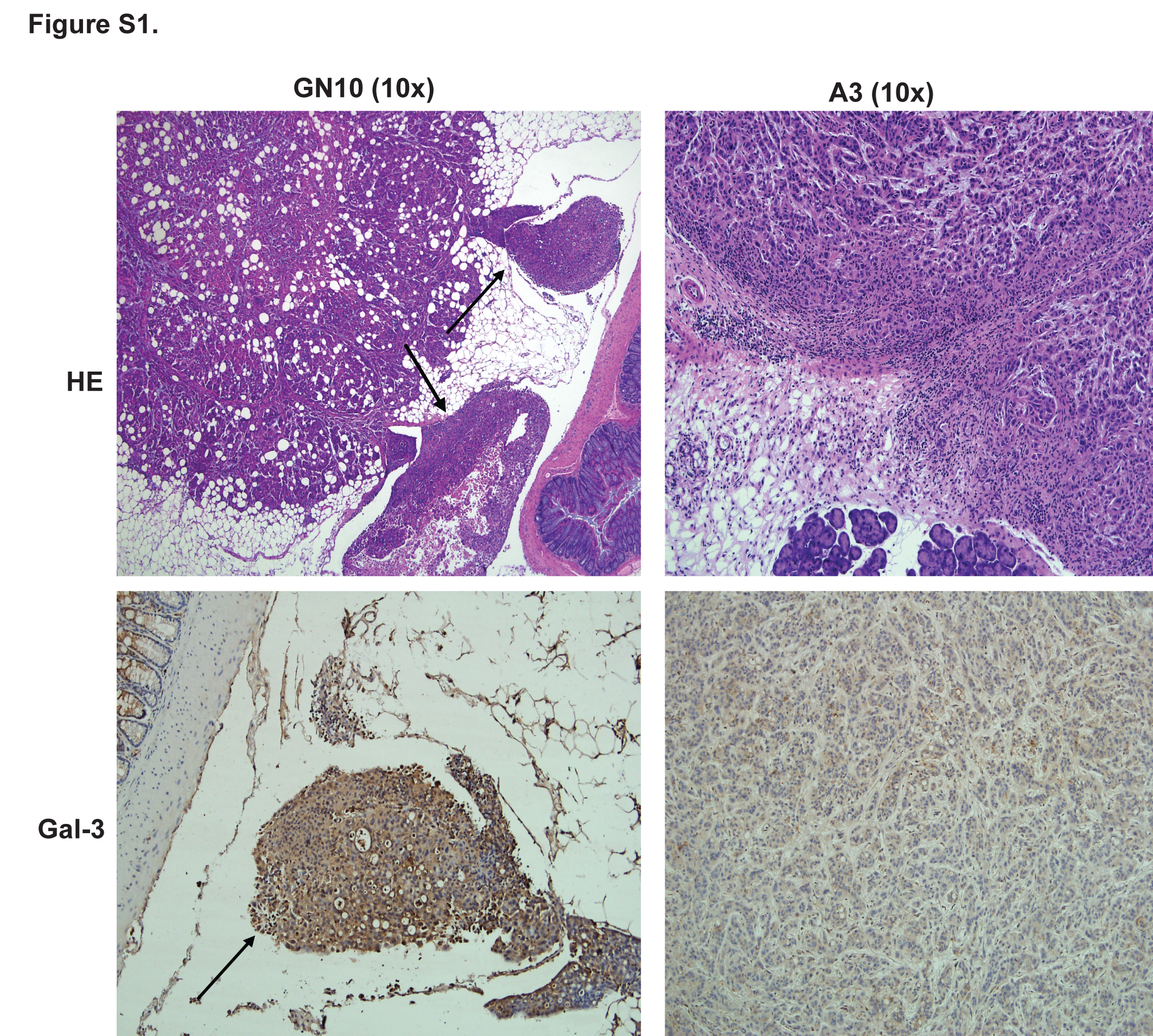

Supplement: Figure S1 — Micrometastases (indicated with arrows) in MPanc96 GN10 mice but not in A3 mice. Representative tumor tissues were stained HE (Upper panel) and Gal-3 IHC (Lower panel) from Mpanc96 Control mice (GN10) and Gal-3 knock down (A3) mice. (TIF) [file pone.0042699.s001.tif]
